# Clinical and imaging characteristics, outcomes and prognostic factors of intraocular foreign bodies extracted by vitrectomy

**DOI:** 10.1038/s41598-023-41105-5

**Published:** 2023-08-29

**Authors:** Xin Liu, Qinzhu Bai, Xiande Song

**Affiliations:** 1 Eye Center, The Second Hospital of Jilin University, #218 Ziqiang Street, Changchun, 130041 Jilin China; 2 Department of Radiology, The Second Hospital of Jilin University, #4026 Yatai Street, Changchun, 130024 China; 3 Department of Ophthalmology, The First Hospital of Qiqihar, #30 Gongyuan Road, Qiqihar, 161005 Heilongjiang China

**Keywords:** Health care, Medical research

## Abstract

To investigate the clinical and computer tomography (CT) features and visual prognostic factors of pars plana vitrectomy (PPV) for management of retained posterior segment intraocular foreign body (IOFB). Medical records of 96 patients with IOFB removed by PPV between July 2017 and June 2021 were retrieved. The medical records, including demographic data, initial and final best corrected visual acuity (BCVA) using standard Snellen chart, characteristics of IOFB, CT findings, and surgical details, were reviewed. Outcome was evaluated according to the final BCVA and prognostic factors were obtained. The mean age was 42.31 ± 12.05 years (range 13–71 years) with 94 males (97.9%) and two females (2.1%). CT was sensitive of IOFB in 93.75% (90 eyes) and the locations were consistent with that found during PPV: 20 foreign bodies were located in vitreous, 6 near ciliary body, and 70 on or in retina. Mean diameter of IOFB removed by PPV is 3.52 mm ± 3.01 mm (range 1–22; median 3), and mean area is 6.29 ± 6.48 mm^2^ (range 0.5–40; median 3), which was statistically associated with the initial VA < 0.1 and endophthalmitis. Endophthalmitis was found in 24 (25.0%) eyes and large wound together with scleral entry site might be related to the endophthalmitis. Visual outcome < 0.1 was associated with relative afferent pupillary defect, initial VA < 0.1, and presence of endophthalmitis. Initial VA ≥ 0.1 was independent predictive factor for a better final BCVA. Relative afferent pupillary defect, initial BCVA < 0.1, and presence of endophthalmitis are poor visual prognostic factors.

## Introduction

Open globe injury is one of the major causes of unilateral blindness, especially in the working-age population. Among them, intraocular foreign body (IOFB) account for 18–40% and the majority of patients are male^1^. The technique of pars plana vitrectomy (PPV) developed rapidly in the past decades and became an important approach for the retained posterior segment IOFB and associated complications. However, the visual outcome may be unsatisfying due to the complex clinical manifestations and complications. It was reported that the visual outcome could be related to some factors such as age, presenting visual acuity, wound length, IOFB size, retinal detachment (RD) and endophthalmitis^2–4^. In this retrospective case study, we aimed to investigate the clinical and computer tomography (CT) features and visual prognostic factors of PPV for managing the retained posterior segment IOFB.

## Materials and methods

### Patients and study design

This is a retrospective study conducted in the tertiary ocular fundus department, Eye Center, the Second Hospital of Jilin University, China. The study was conducted in accordance with the Declaration of Helsinki and was approved by The Ethics Committees of the hospital, which waived the need of informed consent. The medical records of all patients who underwent PPV for the retained posterior segment IOFB between July 2017 and June 2021 were retrieved and details such as age, gender, type of accident, initial and final best corrected visual acuity (BCVA) (Snellen Chart), time of initial injury, characteristics of the IOFB, CT findings, data of primary repair and initial ocular findings, were included. The locations of the entry wound were classified according to the Ocular Trauma Classification Group and categorized into three zones^5^. Patients with anterior segment IOFB, perforating ocular injury, follow-up period less than 1 year, history of previous ocular injury, or low vision from other reasons, were excluded.

All patients received ophthalmological examinations including slit-lamp examination, indirect ophthalmoscopy, and CT at presentation. The orbital CT images were performed with 16-detector CT with a 2-mm axial slice thickness from the frontal sinus level to the infraorbital rim level. All CT images with both the bone and soft tissue windows were reviewed by the same radiologist without knowledge of the patient's information^6^. Visual acuity of counting fingers (CF) was expressed as 0.01 decimal unit; whereas visual acuity of hand movement (HM) and light perception were expressed as 0.002 and 0.001 decimal unit, respectively^3,7^.

### Treatment

Primary suture was carried out in patients with leaking wounds^3^. All eyes underwent 23 or 25 Gauge PPV to remove the foreign bodies with the intraocular magnet or forceps. The surgical data included the site of the foreign body removed, lens surgery, and the type of tamponade. During operations, situation of retina, location and number of IOFB were recorded. RD, proliferative vitreoretinopathy (PVR) and number of additional surgeries during the follow-up period were identified. In all cases, intravitreal vancomycin 1 mg/0.1 mL was injected. In patients with endophthalmitis, systemic antibiotics were used at same time.

### Statistical analysis

Statistical analyses were performed using the SPSS Statistics version 19 (SPSS Inc., Chicago, IL, USA). A chi-squared test or Fisher test for categorical variables was applied to compare the differences among subjects^8^. Univariate logistics regression was applied to evaluate the associations between risk factors and final BCVA^3^. A *P* value of less than 0.05 was considered statistically significant throughout the study.

### Ethical approval

The study was approved by the Ethics Committee of the Second Hospital of Jilin University (2022YanShen97.) and adhered to the Helsinki Declaration at all stages. The Ethics Committee of the Second Hospital of Jilin University waived the need of informed consent.

## Results

Of 281 patients, 282 eyes had ocular injuries associated with IOFB. Among them, 114 cases had posterior segment IOFB.10 cases were excluded due to inadequate data in the medical records, and seven patients with < 6 months follow up period were also excluded. One eye underwent enucleation 2 weeks later because of severe orbital cellulitis and was excluded.

### Epidemiological data, clinical features and ophthalmic findings

**Figure 1:**
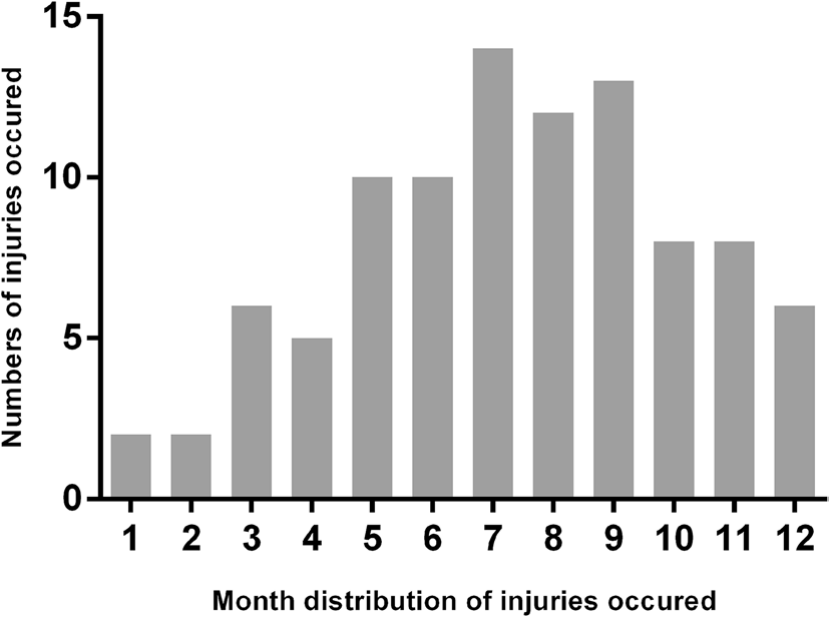
Month distribution of injuries occurred.

Overall, 96 patients with posterior segment IOFB were evaluated in this study, with 94 males (97.9%) and two females (2.1%). Injuries in the right eye were 46 (47.9%) cases and that in the left were 50 (52.1%) cases. The mean age was 42.31 ± 12.05 years (range 13–71 years). The mean duration of follow up was 20.5 months (range 6–253 months). Injuries occur most frequently in July to September (42.71%) as constructing and farming work are most busy and occur less frequently in winter for the cold weather and less work (Fig. 1). Construction work-related injury mechanisms included flying objects from electric pickaxe, grinder and axle in 58 (60.42%) eyes, and was the most common mechanism in this case series. Hammering on nail caused IOFB in 13 (13.54%) eyes and other injuries in 23 (23.96%) cases. The resone for injury was not known in two patients. No goggles for eye protection were applied in this case series. Other demographic data of patients in the study are shown in Table 1.

**Table 1:** Demographic data of patients in the study.

Male	94 (97.9%)
Right eye affected	46 (47.9%)
Mean age	42.31 ± 12.05 (range 13–71 years)
Causes of injury	
Construction work-related injury	58 (60.42%)
Hammering on nail	13 (13.54%)
Other injuries	23 (23.96%)
Unknown	2 (2.08%)

**Figure 2:**
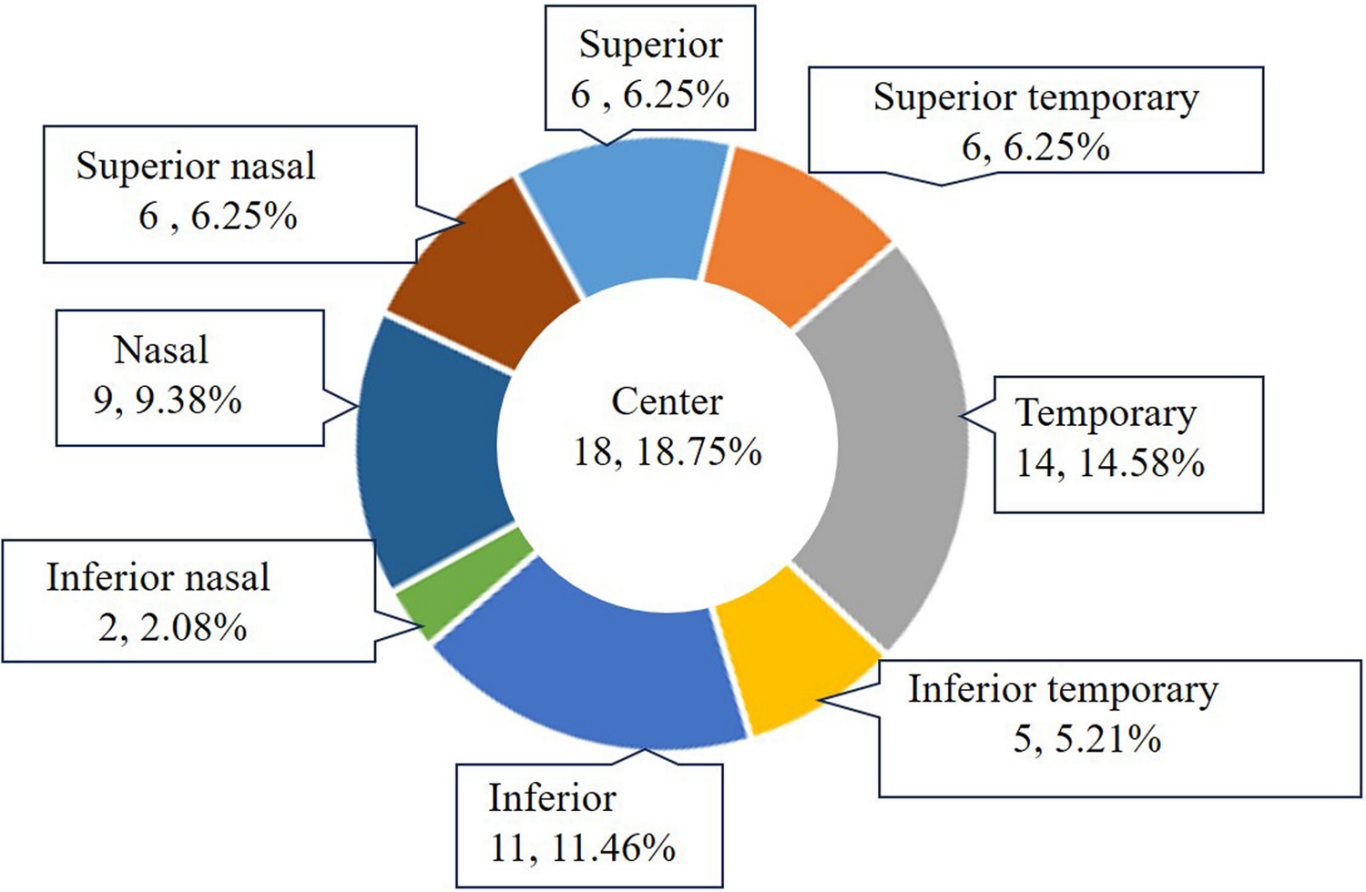
Distribution of entry sites.

Mean length of wound was 2.33 ± 1.69 mm (no sign to 7 mm). Distribution of entry sites in the 78 eyes with signs of wounds was shown in Fig. 2. The retinal injuries, including retinal hemorrhage, retinal blood vessel obstruction, retinal break, and retinal detachment, occurred in 56 (58.33%) patients. The mean time interval from injury to presentation was 108.6 ± 530.25 days (range 0.13–4725 days, median 3 days), and the mean time interval from injury to IOFB removal was 109.69 ± 530.04 days (range 1–4725 days, median 3.5 days). In the 18 eyes with self-healing wounds, mean time interval from injury to presentation was 445.35 ± 1158.41 days, and the mean time interval from injury to IOFB removal was 446.55 ± 1157.94 days. Twelve patients (12.5%) underwent removal of the IOFB within 24 h after the injury, and 65 (67.7%) within 7 days after the injury. Other clinical findings and complications are shown in Table 2.

**Table 2:** Clinical findings and complications.

	Number of eyes	
	n	%
Wound location		
Cornea involved	55	57.3
Central	18	
Peripheral	37	
Limbus involved	15	15.6
Sclera involved	8	8.3
Self-sealing wounds without signs	18	18.8
Wound size		
< 3mm	33	34.4
3–5mm	42	43.8
> 5mm	3	3.1
Self-sealing wounds without signs	18	18.8
Hyphema	7	7.3
Eye contents prolapse	6	6.25
Iris injury	30	31.3
Lens injury	80	83.3
Subluxation	3	3.1
Traumatic cataract	78	81.3
Vitreous hemorrhage	24	25.0
Retinal hemorrhage	14	14.6
retinal blood vessel obstruction	15	84.3
Retinal break	15	15.6
Retinal detachment at presentation	9	9.3
After PPV	3	3.1
Foveal affected	16	16.7
PVR	8	8.3
Endophthalmitis	24	25.0
Siderosis	8	8.3
Glaucoma	3	3.13

*PVR* proliferative vitreoretinopathy, *PPV* pars plana vitrectomy.

24 (25.0%) patients presented with endophthalmitis, while no one developed endophthalmitis after initial management. All of the 24 patients received preoperative systemic antibiotics and intraocular vancomycin injection at primary surgery. Cultures from 13 eyes were sent for microbiological tests before primary surgery and bacterial growth was positive in 3 cases. In clarifying the nature of IOFB with endophthalmitis, 18 from metallic FBs, one from wood, one from fireworks, one from plastic, one from collagen suture, and two from unknown material. There was no statistically significant link between the nature of FB and endophthalmitis development (*P* = 0.188). In the endophthalmitis group, the mean interval from injury to primary surgery was 3.86 days (range 0.25–20, median 2, SD 4.69 days), and from injury to IOFB removal was 4.59 days (range 0.25–20, median 2, SD 4.75 days). A delayed primary surgery or IOFB removal of more than 24 h (*P* = 0.300, 1.000, respectively) showed no statistical correlation with endophthalmitis development. In this cohort, delayed removal often occurs in cases in which the PPV was not available when primary repair applied, or in which the patients were not aware of the injury. Five eyes (62.5%) with trauma on the sclera had endophthalmitis, while 4 eyes (26.7%) on limbus, 14 eyes (25.5%) on cornea, and 1 eye with no sign of trauma had endophthalmitis, the difference is significant (*P* = 0.021). Large wound was associated with occurrence of endophthalmitis: eyes with endophthalmitis had wound length of 3.33 ± 1.66 mm, while those without endophthalmitis had wound length of 1.99 ± 1.56 mm (*P* = 0.001). 1 eye (5.6%) developed endophthalmitis in eyes with self-sealing wound, while 23 eyes (29.5%) had endophthalmitis with visible wounds (*P* = 0.037). None of the other predictors, including nature of IOFB, pre-operative antibiotics, interval before primary surgery or IOFBs removal, primary or secondary removal of IOFB, iris injury, lens injury, VH, or eye contents prolapse, were associated significantly with endophthalmitis (by univariate analysis).

### Features of intraocular foreign bodies and CT findings

The majority of IOFB removed by PPV were metal in 82 eyes (85.5%), stone in three eyes (3.09%), eye lashes in two eyes (2.09%), fireworks in two eyes (2.06%), glass in one eye (1.03%), wood in one eye, collagen sutures in one eye (1.04%), stone with eyelash in one eye (1.03%), and unknown material in three eyes (3.09%). Among the metal foreign body injuries, 78 eyes had magnetic foreign bodies, whereas 4 eyes had nonmagnetic foreign bodies, explicitly copper, aluminum, and titanium. Single IOFB was found in 89 eyes (92.71%), two foreign bodies were in 4 eyes (4.17%), three foreign bodies in 2 eyes (2.08%), and small and massive foreign bodies in one eye (1.04%). 20 (20.83%) eyes had foreign bodies located in vitreous body, 65 (67.71%) on retina, 6 (6.25%) on ciliary body. Other 5 eyes experienced foreign bodies on retina, and at the same time on anterior part of eye or vitreous. In the IOFB removed by PPV, 20 FBs were located in vitreous, 6 near ciliary body, and 70 on or in retina. Among them, 8 eyes (8.33%) experienced IOFB impact on the macula. Mean diameter of IOFB removed by PPV is 3.52 mm ± 3.01 mm (range 1–22; median 3), and mean area is 6.29 ± 6.48 mm^2^ (range 0.5–40; median 3). The analysis of variance showed that the larger IOFB dimension was statistically associated with the initial BCVA < 0.1 (*P* = 0.004, *P* = 0.008 for diameter and area, respectively), larger wound size (*P* = 0.001 for area), endophthalmitis(*P* = 0.034, *P* = 0.007 for diameter and area, respectively), and macular trauma (*P* = 0.040 for diameter).

**Figure 3:**
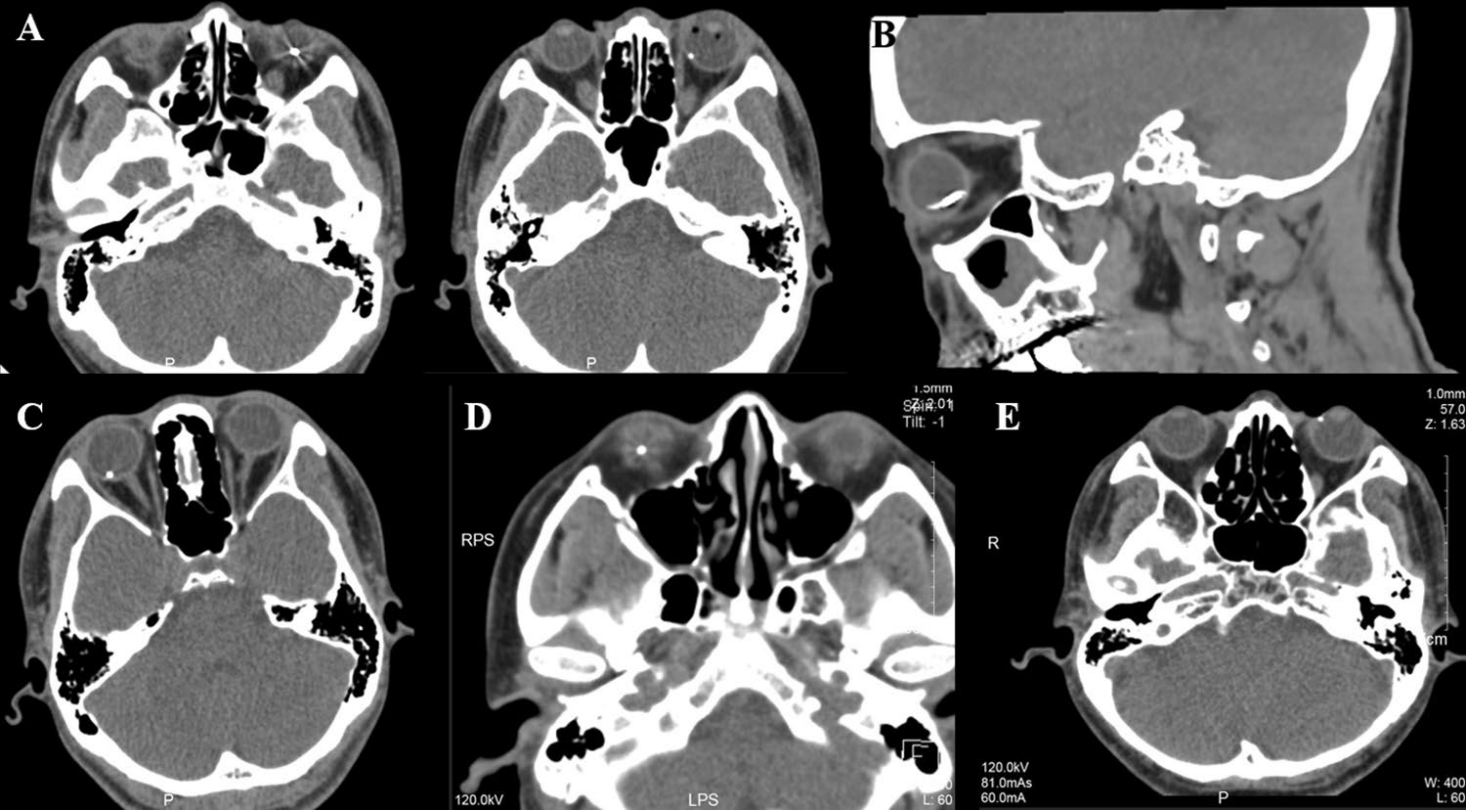
(**A**) Two foreign bodies in one eye on different layers of CT; (**B**) large foreign body of 15 mm × 3 mm; (**C**) foreign body lay on retina; (**D**) foreign body in vitreous body; (**E**) foreign body near ciliary body.

CT was performed preoperatively in all eyes and sensitive of IOFB in 93.75% (90 eyes) of cases, and locations of IOFB were same as that found during PPV (Fig. 3). For multiple foreign bodies in 7 patients, CT could only be shown in one patient. IOFBs failed to reveal in 3 patients who had eye lashes on retina, in 1 patient had tiny iron dust near ciliary body, in 1 patient had wood and sawdust, and in 1 patient had collagen suture. Slit-lamp examination with indirect ophthalmoscopy fundus examination could find IOFB in 31 eyes (32.29%). This was because of traumatic cataract, severe reaction or hyphema in anterior chamber, endophthalmitis, or VH. Comparison of ophthalmic findings in ophthalmic examination and CT was listed in Table 3, the sensitivity of these ophthalmic changes was varying from 28.57 to 62.50% since the structure of eye is subtle and complex and difficult to distinguish. The specificity of collapsed anterior chamber, hyphema, lens trauma, siderosis and retinal detachment was 100% in this case series, and of vitreous hemorrhage was 95.89%. The size of the IOFB in each eye on CT image was measured using digital calipers on the CT scan and later compared with the size of the removed IOFB. The ratio of sizes on the CT scan to actual IOFB sizes varied from 0.69 to 3.5 (mean 1.46).

**Table 3:** Comparison of ophthalmic findings in ophthalmic examination and CT.

	Number of eyes		Sensitivity (%)	Specificity (%)
	Ophthalmic examination	CT		
IOFB				
Detection of IOFB	–	90	93.75	100
Locations of IOFB	–	90	100	100
Multiple foreign bodies	7	1	14.29	100
Ophthalmic findings	3	3	100	100
Collapsed anterior chamber				
Hyphema	7	2	28.57	100
Lens trauma	80	36	45.0	100
Vitreous hemorrhage	23	11	47.83	95.89
Retinal detachment	9	3	33.33	100
Siderosis	8	5	62.50	100

Sensitivity = TP/(TP + FN), specificity = TN/(TN + FP).

*TP* true positive, *FP* false positive, *FN* false negative, *TN* true negative, *CT* computer tomography.

### Surgical management and visual prognosis

For surgical management, primary globe repair was performed prior to removal of the IOFB in 38 eyes (39.6%), primary globe repair combined with PPV and IOFB removal as a single procedure was used in 7 eyes (7.3%). PPV without primary globe repair was used for patients with a self-sealing wound (50 eyes [52.1%]); one eye (1.03%) experienced primary globe repair combined with magnetic removal of one foreign body in the vitreous, and PPV removal of another foreign body. IOFB were removed with a magnet in 75 eyes (78.1%) or intraocular forceps in 21 (21.9%). IOFB were removed through the sclera in 77 eyes (80.2%) and through the trans-corneal site in 19 eyes (19.8%). Crystalline lens removal with or without intraocular lens insertion was performed in 65 eyes (67.71%) and among them, cataract extraction was performed at the time of initial globe closure, IOFB removal and vitrectomy in 60 eyes, during silicone oil removal in 3 eyes, and after PPV in 2 eyes. IOL was implanted at the end of surgery or a later stage in 47 eyes, while 18 eyes remained aphakic as they had extensive ocular damage with low potential for visual improvement. Overall, 6 eyes required additional vitrectomy and retinal surgery: three eyes for post-operative retinal detachment, one eye for retina break, one eye for macular membrane removal, and one eye for post-operative PVR. Silicone oil tamponade was used in 47 eyes (49.0%), including 2 eyes which developed retinal detachment after primary PPV, and gas tamponade in 20 eyes (20.8%). Anatomical success was achieved in all patients although some of the patients had silicone oil tamponade at last visit.

**Figure 4:**
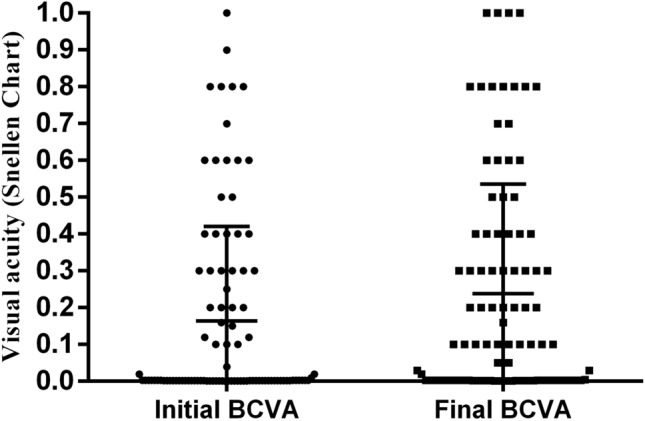
The initial and final visual acuities distribution. *BCVA* best corrected visual acuity.

The initial and final visual acuities are presented in Fig. 4. 59 (61.5%) patients initially presented with a Snellen visual acuity (VA) < 0.1. After surgeries, 20 (20.8%) eyes had a final visual outcome of 0.5 or better; however, visual outcomes worse than 0.1 were found in 44 (45.8%) eyes. For poor visual outcome, univariate analysis showed that presence of relative afferent pupillary defect (RAPD), initial VA < 0.1, and presence of endophthalmitis (*P* = 0.006, < 0.001, and = 0.044) were the significant associated predictors (Table 4). Stepwise logistic regression analysis revealed that the initial VA (*P* = 0.001; odds ratio 8.69; 95% CI 3.13–24.12) was an associated predictive factor. For good visual outcome, absence of RAPD and initial VA ≥ 0.1 (*P* = 0.032, P < 0.001) were the significant associated predictors (Table 4). Stepwise logistic regression analysis revealed that the initial VA (*P* = 0.001; odds ratio 7.364; 95% CI 2.39–22.73) was an associated predictive factor of visual outcome ≥ 0.5.

**Table 4:** Frequency and significance of the prognostic factors for visual outcomes.

Variable	Final BCVA ≥ 0.1 n (%)	*P* value	Final BCVA ≥ 0. 5 n (%)	*P* value
Age ≥ 50 years				
Yes	13 (43.3)	0.151	3 (10.0)	0.078
No	39 (59.1)		17 (24.8)	
Wound site				
Cornea	33 (60.0)	0.480	12 (21.8)	0.761
Limbus	9 (60.0)		2 (13.3)	
Sclera	3 (37.5)		1 (12.5)	
No signs	8 (44.4)		5 (21.8)	
Hyphema				
Yes	3 (42.9)	0.697	0 (0)	0.339
No	50 (56.2)		20 (22.5)	
Iris injury				
Yes	19 (63.3)	0.224	8 (26.7)	0.343
No	33 (50.0)		12 (18.2)	
RAPD				
Yes	12 (35.3)	0.006	3 (8.8)	0.032
No	40 (64.5)		17 (27.4)	
Lens injury				
Yes	38 (47.5)	0.233	63 (78.8)	0.822
No	5 (31.3)		13(81.3)	
Vitreous hemorrhage				
Yes	14 (58.3)	0.636	4 (16.7)	0.562
No	38 (52.8)		16 (22.2)	
Initial VA ≥ 0.1				
Yes	31 (83.8)	< 0.001*	15 (40.5)	< 0.001^#^
No	22 (37.3)		5 (8.5)	
RD at presentation				
Yes	3 (37.5)	0.291	1 (12.5)	0.680
No	49 (56.3)		19 (12.8)	
PVR				
Yes	4 (50.0)	0.805	2 (25.0)	0.762
No	4 (54.5)		8 (20.5)	
Endophthalmitis				
Yes	9 (37.5)	0.044	2 (8.3)	0.082
No	44 (61.1)		18 (25.0)	
Siderosis				
Yes	3 (37.5)	0.463	3 (37.5)	0.357
No	49 (55.7)		17 (18.3)	
IOFB size (mm)				
≥ 5	7 (53.8)	0.980	2 (15.4)	0.603
< 5	45 (54.2)		18 (21.7)	
IOFB location				
Retina	35 (52.2)	0.567	13 (19.4)	0.842
Vitreous	3 (50.0)		1 (16.7)	
Ciliary body	15 (65.2)		6 (26.1)	
IOFB extraction ≥ 24h				
Yes	47 (56.0)	0.573	18 (21.4)	0.704
No	5 (41.7)		2 (16.7)	

*BCVA* best corrected visual acuity, *RAPD* relative afferent pupillary defect, *RD* retinal detachment, *PVR* proliferative vitreoretinopathy, *IOFB* intraocular foreign body.

*Stepwise logistic regression analysis revealed that the initial VA ≥ 0.1 was independent predictive factor for final BCVA ≥ 0.1 (P = 0.001; odds ratio 8.69; 95% CI 3.13–24.12).

^#^Stepwise logistic regression analysis revealed that the initial VA ≥ 0.1 was independent predictive factor for final BCVA ≥ 0.5 (P = 0.001; odds ratio 7.364; 95% CI 2.39–22.73).

## Discussion

Our patients were 42.31 ± 12.05 years on average and included a total of 94 males (97.9%), which was similar to the demographic characteristics of other reports^3^. Young men are more predisposed to eye injuries as they participate in more risky activities and manual labor. The most common factors associated with eye injuries were not wearing goggles and metal hammering^3^. In our case series, most of the accidents happened at the workplace, especially when grinder or hammer was occasionally used and they thought wearing goggle was troublesome. Therefore, use of protection should be emphasized for working young people. The entry site on cornea accounted for most of the wound in 55 eyes (57.3%), and this was similar to that in other reports^1,2^. Self-sealing wounds without signs were found in 18 patients (18.8%) who presented in hospital until blurred vision or red eye for a long time.

The incidence of infectious endophthalmitis in our case series was 25.0% which was high than that in reports of open globe injuries with retained IOFB (0–20%)^2,9^, but similar to that of retained IOFB requiring PPV (4.8–28.6%)^2,10–12^. Larger wound and IOFB, and wounds on sclera were found to be associated with endophthalmitis in this study. In some other studies, endophthalmitis was also found to be related to wound length > 3 mm^13^. Endophthalmitis developed less in eyes with self-sealing wound with no signs in this study, which was rarely reported before. There may be no apparent symptoms and no endophthalmitis, so trauma is ignored and presentation is delayed. The mean time interval from injury to presentation and that from injury to IOFB removal both varied from few hours to 13 years, and this was not correlated to endophthalmitis occurrence in our study. Some studies have shown that delayed removal of IOFB with PPV may end with poor visual and development of infectious endophthalmitis, because prompt removal decreased the time that can be used for microorganisms’ proliferation on IOFB and cleared the vitreous which is a good culture medium for microorganisms^1,14^. More recent studies have suggested that delayed IOFB removal may not increase risk of endophthalmitis or other complications with high rates of prophylactic intraocular antibiotic usage^14–16^. Delays in IOFB removal may be necessary in patients with severe corneal edema and inflammatory reaction. If primary removal is not possible, prompt globe closure with sufficient antibiotics is suggested.

Single IOFB was found in 89 eyes (92.71%), two foreign bodies were in 4 eyes (4.17%), three foreign bodies in 2 eyes (2.08%), and small and massive foreign bodies in one eye (1.04%). Multiple FB were rarely reported in literatures, but the missing FB may cause severe inflammation, toxicity, and risk for endophthalmitis^17^. The eye with FB located in anterior part of eye may also have FB in posterior eye, especially those with negative FB on CT which may be neglected after primary removal of anterior IOFB or one posterior IOFB. In our cohort, 7 eyes with multiple IOFBs needed 1–3 (average: 1.86) times of surgeries to remove the FB, among them 2 eyes had two times of PPV to remove the FB. We suggested fundus examination, CT, MRI or B-scan applied after successful removal of FB, especially those who still have severe inflammation or uncontrollable endophthalmitis. Vitrectomy may be an ultimate approach if all of the examination result is negative in these eyes, as some foreign bodies are difficult to be revealed by imaging methods. Eye lashes were found in three eyes in this study and caused severe inflammation, until vitrectomy was applied.

CT is useful in detecting foreign bodies with a sensitivity ranging from 65% (for a foreign body < 0.06 mm^3^) to 100% (for foreign bodies > 0.06 mm^3^)^18^ and is generally considered to be the gold standard for IOFBs^19^. Moreover, CT has big advantage in detecting and localizing rigid intraocular metallic, glass, and stone foreign bodies, especially in searching for potential unseen IOFB^20^. In this case series, the sensitive of IOFB detected by CT is 93.75% while that by lit-lamp is 32.29%. In the 6 eyes of no signs of IOFB on CT, 3 patients had eye lashes on retina, 1 patient had tiny iron dust near ciliary body, 1 patient had wood and sawdust, and 1 patient had collagen suture. All of these were difficult to be detected by CT as the features of small or non-magnetic, and patients had PPV to find out them. A relatively low rate of detection of IOFB by lit-lamp was because of traumatic cataract, severe reaction or hyphema in anterior chamber, endophthalmitis, or VH. Meanwhile, the FB location in CT was accurate in all eyes which could help in clinical decision making in methods of foreign body removal. In cases with IOFB embodied in retina, magnetic removal without PPV might cause RD, while in PPV, retinal photocoagulation or other approaches could be done first to provide the retinal traction. Besides IOFB, CT could also find some other signs of ocular injury and be helpful in some cases unable to cooperate with eye examination with a high specificity, such as anterior chamber collapse, hyphema, lens trauma, vitreous hemorrhage, retinal detachment, and siderosis. For VH, the specificity was 95.89%, which might be influenced by the radiology of foreign body as CT is a technique of density imaging. Preoperative estimation of the IOFB size and location by CT scan was also helpful in the decision-making of IOFB extraction. When the size of the IOFB was estimated to be large, the enlarged sclera incision or another limbus incision wound would be used. It often occurs that the size of metallic IOFBs on the soft tissue window setting would be enlarged, while it is almost the real size in the bone window setting^21,22^. This study measured the size of the IOFB on bone windows of CT scan. The ratio of real sizes on the CT scan to actual IOFB sizes varied from 0.69 to 3.5 (mean 1.46), and this was in agreement with prior study^6,23^. The overestimation of the size is due to artifacts caused by metal IOFB. Irregular FBs may cause an underestimation of the size. Adjustment of the window width and center values on the CT was suggested to reduce artifacts and obtain more precise sizes^6^.

Good final visual outcome (VA of 0.5 or better) was reported in 30–71% of patients^2,12,15^. In this study, PPV and IOFB removal helped 20 (20.8%) patients to gain favorable visual outcome (VA ≥ 0.5). Poor final visual outcome (VA of less than 0.1) was reported previously in 17–50% of patients^2,15^, and it was 45.8% in the presented study.

The visual outcome was not quite satisfying as reported, and this may relate to the late referral at our tertiary care center and severe damage which could not be managed by other hospitals. Prognostic factors of worse visual outcome included poor initial VA (*P* < 0.001), presence of RAPD (*P* = 0.006), and presence of endophthalmitis (*P* = 0.044), and that of good visual outcome included absence of RAPD (*P* = 0.032) and initial VA ≥ 0.1 (*P* < 0.001). Initial BCVA was an independent prognostic factor throughout our study, and this is congruous with most of the previous studies^3,4,16,24–28^, mainly because it reflects ocular damage degree at presentation. Severe VH or traumatic cataract might influence a lot on initial VA and could be managed well by surgery, and this may be the reason why in some studies initial BCVA was not correlated with a poor visual prognosis^2,29,30^. Presence of RAPD was statistically significant correlated to the poor final visual outcome, which was also reported by Choovuthayakorn et al.^31^. Presence of RAPD implies a severe damage of eye, especially the retina, and when this sign is found, the visual outcome might be compromised. Consistent with some previous reports, endophthalmitis was corelated to a poor final VA^3,13,32^. Endophthalmitis could cause a devastating damage to intraocular structures especially to retina. Among the 24 patients with endophthalmitis, 6 (50.0%) had RD at presentation or during follow-up, whereas in the 72 cases without endophthalmitis, only 6 eyes had RD (*P* = 0.033). Meanwhile, retina breaks and blood vessels occlusion occurred more in eyes with endophthalmitis, 7 eyes (46.7%) and 8 eyes (50%) respectively, which were statistically significant (*P* = 0.035, *P* = 0.023).

Pre-operative and post-operative RD are found to be associated with high risk of poor visual outcome^2,3,26,33^. Our study did not demonstrate a correlation between the RD and visual prognosis. A recent study revealed that if the detached retina was reattached without macular involvement, the final VA could be better^30^. Several authors also described scleral/corneoscleral entry^3,31^ and IOFB ≥ 3 mm^15,29,33–36^ to be important predictive factors for the poor visual outcome. However, our results did not show a correlation between the visual outcome, IOFB size and entry site. The location of the entry site was not a prognostic factor for the visual outcome in some series either^20,37^.This could be explained by the fact that the penetrating site and place on retina of foreign body is not usually the largest edge of the foreign body.

This case cohort had one of the largest number of patients as well as a relatively long-term post-operative information which was rare in the previous studies, adding to the strength of our results. Nevertheless, the study had some limitations. These included its retrospective design with all data collected from medical charts, wide ranges of follow-up periods, and the variable repairing surgical skills from the different primary surgeons, which might have introduced important bias.

## Conclusions

Patients with posterior segment IOFB can have a wide variety of presenting features and the restrained visual prognosis. This cohort of patients with posterior segment IOFB removed by PPV have gained acceptable visual results, despite a longer time interval between injury and surgery or retinal damage, even detachment. The poor initial VA, presence of RAPD and endophthalmitis contributed to significant visual morbidity in this study. Endophthalmitis was significantly associated with a scleral entry wound or a large size of the IOFB. If primary removal of IOFB is not possible, prompt globe closure with sufficient antibiotics is suggested to reduce the occurrence of endophthalmitis and thereby improve visual outcome. IOFB more than one is relatively rare but could cause a lot of troubles, including but not limited to more operations, ophthalmologists should stay vigilant in handling injuries with IOFB.

## Data Availability

The datasets generated during and/or analyzed during the current study are available from the corresponding author on reasonable request.
